# A qualitative study of stakeholders’ perceptions towards the implementation of the National Health Insurance Scheme in Zambia: equity and financial sustainability in question

**DOI:** 10.1093/heapol/czag074

**Published:** 2026-05-25

**Authors:** Joseph Kazibwe, Jesper Sundewall, Felix Masiye, Lucy Owusu, Phuong Bich Tran, Chitalu M Chama-Chiliba, Björn Ekman, Pia Svensson

**Affiliations:** Department of Clinical Sciences Malmö, Lund University, Malmö, Sweden; Department of Clinical Sciences Malmö, Lund University, Malmö, Sweden; HEARD, University of KwaZulu-Natal, Durban, South Africa; Department of Economics, University of Zambia, Lusaka, Zambia; Health Service and Population Research Department, King’s College London, London, United Kingdom; Nuffield Department of Primary Care Health Sciences, University of Oxford, Oxford, United Kingdom; Centre for Economic and Social Research, University of Zambia, Lusaka, Zambia; Department of Clinical Sciences Malmö, Lund University, Malmö, Sweden; Department of Clinical Sciences Malmö, Lund University, Malmö, Sweden

**Keywords:** National Health Insurance, health financing, stakeholder perceptions, policy implementation, enablers, challenges, low- and middle-income countries, Zambia

## Abstract

Low- and middle-income countries are expanding health insurance to enhance financial protection. However, there is scarcity of evidence on the stakeholder perceptions towards health insurance. This study explores stakeholders’ perceptions towards the implementation of the National Health Insurance Scheme (NHIS) in Zambia. This qualitative study is based on 21 in-depth interviews with key stakeholders involved in the NHIS implementation in Zambia. We used qualitative content analysis to describe and interpret the manifest and latent meaning of transcribed interviews. The analysis yielded three overarching themes: (i) politics can make or break the scheme; (ii) equity in question: winners and losers; and (iii) pointing at the need for financial sustainability of the NHIS. The themes are supported by 6 subthemes and 18 data categories. Participants perceived the scheme as having contributed to improved quality of care and offering a generous health benefit package. However, implementation is perceived to be hindered by political interference, limited use of empirical evidence, weak institutional coordination, and exclusion of key stakeholders from decision-making processes, low enrolment, operational inefficiencies, inequities and a benefit package misaligned with available resources. The Zambia NHIS is perceived as a promising initiative for expanding access to health and enhancing financial protection. However, it faces numerous challenges ranging from limited enrolment among the poor, vulnerable and informal sector populations to concerns about financial sustainability. Addressing these issues is essential to realize the scheme’s potential benefits.

Key messages

- National Health Insurance Scheme enjoys public support despite the perceived challenges it faces.
- The implementation of the National Health Insurance Scheme has led to disparities in access and quality of care, especially between urban and rural areas and among socio-economic groups.
- The scheme faces financial challenges where the total claims for reimbursements are more than the revenue. There are concerns of the scheme having an overly generous benefit package and insufficient premium contributions, especially from informal sector members.
- Stakeholders perceive that political interests influence the operations of the National Health Insurance Authority, often undermining technical decision-making and transparency. They also highlight a lack of systematic consultation and limited use of available evidence in shaping policy and operational decisions.

## Introduction

Public health insurance, which the World Health Organization (WHO) recommends as a strategy to enhance access to care while protecting individuals from catastrophic health expenditure (CHE), decouples access to healthcare from the ability to pay at the point of service (World Health Assembly 2005). Out-of-pocket (OOP) health expenditure refers to the direct payments households make for healthcare goods and services from their income or savings at the point of use (Eurostat 2025). Although OOP spending is typically voluntary (World Health Organisation 2025), it can undermine a household’s ability to meet basic needs or limit access to other essential goods and services, resulting in financial hardship. A household is considered to face financial hardship when its health expenditure exceeds 40% of its discretionary budget, defined as total consumption or income after accounting for the cost of basic needs (World Health Organisation 2025). Financial strain from OOP payments can also be assessed through CHE, which occurs when OOP health spending exceeds 10% or 25% of total household consumption or income (World Health Organisation 2025). Public health insurance schemes are particularly relevant in settings where out-of-pocket payments often pose significant barriers to healthcare utilization and financial protection.

Evidence on the impact of public health insurance in low- and middle-income countries (LMICs), however, remains mixed. While many studies report increased utilization of specific health services following the introduction of insurance schemes, others highlight limited or uneven effects (Erlangga et al. 2019, Kazibwe et al. 2024). Systematic reviews have noted the difficulty of comparing findings across contexts due to variations in health insurance scheme design, enrolment strategies implementation, and local health system capacities (Erlangga et al. 2019, Kazibwe et al. 2024). These inconsistencies underscore the importance of analysing processes and policy contexts of health insurance schemes to understand what factors contribute to effective and equitable implementation.

Zambia’s health financing system has undergone multiple health reforms to improve access, equity, and financial protection (Chansa et al. 2008, Chitah et al. 2018, Osei Afriyie et al. 2025). Initiatives such as the removal of user fees in primary care were intended to reduce financial barriers, yet CHE has persisted at significant levels. In 2017, the prevalence of CHE among households remained high at ∼5.7% (Zambia Statistics Agency 2016), with a marked rural-urban disparity. For example, Lusaka Province (an urban province) had the lowest CHE prevalence (3.6%), and Western Province (a rural province) had the highest (9.3%; Zambia Statistics Agency 2016). In addition, 11% of households with a recent illness struggled to pay for healthcare by borrowing money or selling their property, a sign of financial hardship (Kaonga et al. 2019).

Zambia faces health system financing challenges of insufficient funding and inefficient utilization of resources for health. The government’s current health expenditure has been generally constant over the years as a percentage of gross domestic product (GDP), with donors contributing close to 50% of the current health expenditure (Ministry of Health Zambia 2023). Several inefficiencies have been reported within the health system (Masiye et al. 2006, Chansa et al. 2008, Achoki et al. 2017) and there is a growing interest to address them (Ministry of Health Zambia 2017). External funding for health has been volatile and at times unpredictable, disrupting health programmes. Furthermore, the country has been constrained by growing inflation and increasing domestic and external debt, which limits the ability to increase government funding for health (Ministry of Health Zambia 2023, Musokotwane 2023, Stein and Chitonge 2025).

Zambia’s health system continues to confront major financing difficulties, characterized by inadequate funding levels and inefficient use of available resources. Government current expenditure on health has remained relatively stable over time as a share of the country’s GDP, while external partners finance nearly half of current health spending (Ministry of Health Zambia 2023). Numerous studies have highlighted inefficiencies within the health sector (Masiye et al. 2006, Chansa et al. 2008, Achoki et al. 2017), prompting increasing efforts to address these challenges (Ministry of Health Zambia 2017). However, reliance on external funding has proved problematic due to its volatility and unpredictability, which at times disrupts health programmes. The country’s capacity to increase domestic financing is further constrained by rising inflation and an increased burden of internal and external debt, limiting fiscal space for additional government investment in health (Ministry of Health Zambia 2023, Musokotwane 2023, Stein and Chitonge 2025).

In response, the government enacted the National Health Insurance Act No. 2 of 2018, establishing the National Health Insurance Scheme (NHIS). NHIS is a health insurance scheme with a goal to provide universal access to quality health care services in accordance with the act, and has been operational since October 2019. The act provides for the establishment of the National Health Insurance Management Authority (NHIMA), a corporate body that operates and manages the Scheme (Republic of Zambia 2018). The NHIS was set up with both contributory and non-contributory membership models entitled to a predetermined health benefit package (a set of health services that NHIS members can access) (National Health Insurance Management Authority 2022). The contributory membership model is where an NHIS member is required to periodically (usually monthly) pay a contribution to the scheme in the form of predetermined premiums, while the non-contributory model is where NHIS members are exempt from making contributions to the scheme. This reform (introduction of NHIS) was meant to align with the universal health coverage (UHC) commitments and WHO recommendations.

Membership of the NHIS has shown an increasing trend from 713 952 members as of 2020 to about 1.87 million active members in 2022. Most of the membership is made up of individuals from the formal sector (60.1%). The majority of the members are male (67.2%; ILO 2023). NHIMA has accredited over 450 health facilities across Zambia, including public, private for-profit, and private not-for-profit health providers (National Health Insurance Management Authority 2025).

Introducing national health insurance in a health system is a complex and difficult process shaped by politics, institutional capacity, financing arrangements, and stakeholder buy-in (Houeninvo et al. 2022, Jamal et al. 2022). Policy implementation takes time and hardly goes exactly as planned (Campos and Reich 2019, Buse et al. 2024, Public Health Agency of Sweden 2024). Implementation of a policy will inevitably encounter challenges and usually evolves to address them. In Zambia, the NHIS represents both a policy innovation and an institutional challenge, as its rollout requires changes to provider payment systems, information systems, and benefit administration. While quantitative evaluations can assess the impact of the NHIS on, e.g. service utilization and financial protection, they often provide limited insights into the complex policy processes, operational bottlenecks and adaptive strategies that shape these outcomes.

Until now, there is limited understanding of how stakeholders perceive the implementation of the NHIS. Despite being a major health reform, relatively few studies have investigated the NHIS in Zambia (Sinjela et al. 2022, Osei Afriyie et al. 2023b, 2025). Osei Afriyie et al. (2025) studied the process of development of the scheme up to 2018, but no study has explored the perceptions of key stakeholders on the implementation of the NHIS since it became operational. Understanding these perspectives is critical for identifying context-specific enablers and barriers, informing adjustments, and strengthening implementation. Therefore, this study aims to explore the perceptions of stakeholders towards the implementation of the NHIS in Zambia. The study focuses on some of the challenges and enabling factors that have influenced the implementation of the Zambian health insurance programme thus far. By addressing this gap, this study contributes to the policy debates and broader literature on the political economy and implementation of national health insurance in LMICs.

## Materials and methods

### Study design

We applied a qualitative study design to explore perceptions of key stakeholders on the implementation of health insurance in Zambia. Information was collected through in-depth semi-structured, key informant interviews with participants that are directly or indirectly involved in implementing NHIS in Zambia. Participants were classified as directly involved in the implementation of the NHIS if they took part in the day-to-day management and operations of the scheme, such as NHIMA managers and health practitioners. Those considered indirectly involved were individuals who could influence how the scheme functions but were not engaged in its daily operations, e.g. academicians, funders and civil society. The interviews were analysed using qualitative content analysis with an inductive approach (Graneheim and Lundman 2004). The study follows the standards for reporting qualitative research by O’Brien et al. (2014).

### Setting

The study was carried out in Zambia, a lower middle-income country with a population of ∼20 million people, of which the majority (54%) reside in rural areas. Zambia has high poverty levels, with 47.9% of the population categorized as multidimensionally poor, while 23.9% of the population are classified as vulnerable to multidimensional poverty (United Nations Development Programme 2023). This study was not limited to a specific geographic area within Zambia; it was conducted at a national level to explore stakeholders’ perceptions of the implementation of the NHIS. However, most of the participants were based in urban areas, as many key institutions in Zambia have their head offices located in the capital city, Lusaka.

### Sampling strategy

We used the purposive sampling method to identify and recruit relevant key informants with in-depth knowledge and experience relevant to the implementation of the NHIS. Participants were selected to reflect diverse stakeholder groups involved in the scheme, with key informants drawn from institutions central to NHIS implementation. Formal invitation letters were sent to the identified institutions, which then nominated individuals deemed most appropriate to participate in the study based on their roles and expertise.

The final sample included 21 participants from the central government, local government, the scheme managers, health practitioners’ councils, academicians/researchers, funders and civil society (details provided in Supplemental material S1).

### Data collection method, instruments and technology

The data collection was done through key informant interviews. An interview guide (Supplemental material S2) was developed and piloted prior to its use. The interview guide contained a set of open-ended questions meant to direct the discussions between the key informant and the interviewer. The questions covered a range of NHIS implementation areas, including aims, organization, benefit package, reimbursement, enrolment, and accreditation of providers. The interviews took place in Zambia and lasted on average 60 min, ranging from 34 to 120 min. The interviews were conducted in English by J.K., recorded using a voice recorder and supplemented with notes. Member checking was conducted on six randomly selected participants.

### Data processing and analysis

The recorded interviews were transcribed to text and uploaded to NVivo 14 for analysis following inductive content analysis. The analysis began with familiarization, involving repeated reading of transcripts and listening to interview recordings. Meaningful units (Graneheim and Lundman 2004) were identified based on manifest content and coded. These codes were then grouped into categories, from which subthemes were developed through latent analysis, ultimately leading to the construction of overarching themes.

### Researcher characteristics and reflexivity

The study was conducted by a team of researchers with extensive experience with the Zambian context. The team members bear different characteristics and are representative of different sexes (half of the team is female), ages, and career stages. The team is comprised of two early career researchers (PhD students) in health economics and financing, five senior researchers in health economics and health systems, and one senior researcher in social medicine and qualitative methodology. Three members are based in Zambia, three are based in Sweden, one in the UK, and one in Ghana. The diversity of the team members enhanced the objectivity of the study by curtailing preconceived bias in data collection, analysis, and reporting of the lead researcher. Contextual familiarity facilitated stronger rapport with interviewees, enhancing data quality.

### Ethical considerations

Ethical approval was granted by the Biomedical Research Ethics Committee of the authors’ institute (reference number 6227-2025). The study participants were asked for written consent spelling out their rights, benefits, and likely harms of participating in the study prior to the interview. The interview recordings were anonymized including erasing their professional roles and other identifiers. The anonymized information collected, recordings and transcripts were kept confidential and stored on a secured cloud storage service hosted by the authors’ institute.

## Results

**Figure 1:**
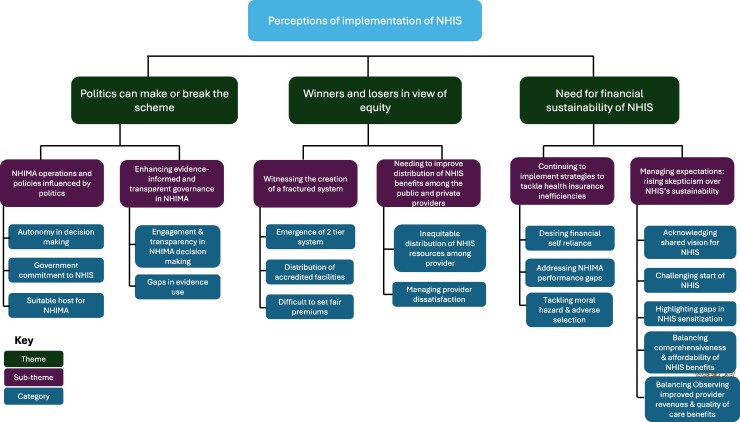
Diagram showing the themes, subthemes and data categories in this study.

The analysis yielded three overarching themes: (i) politics and political interest can make or break the scheme, (ii) equity in question: winners and losers, and (iii) pointing at the need for financial sustainability of NHIS. These themes were elaborated through a total of 6 subthemes that emerged from the 18 data categories, as shown in the diagram below (Fig. 1).

### Theme 1: politics can make or break the scheme

This theme reflects the perceived pivotal role of political dynamics and interests in shaping the success or failure of the scheme. The theme yielded two subthemes described below.

#### Subtheme 1: the National Health Insurance Management Authority operations and policies are perceived to be influenced by political interests and need more support and direction

This subtheme showcases participants’ belief that politics is an integral part in the implementation of the NHIS. It highlights the need to balance greater autonomy within the scheme with sustained political support.

##### Seeing the Ministry of Health as a strategically and technically suitable host for the National Health Insurance Management Authority

NHIMA’s organizational and governance structure was described to be influenced by change of government due to opposing conceptual understanding of health insurance and political interest. NHIMA is attached to the Ministry of Health (MoH; host) to which NHIMA reports. The host ministry is meant to oversee NHIMA’s performance. Participants exclaimed that due to a change in government in 2021 following an election, NHIMA was moved from the MoH to the Ministry of Labour and Social Security (MoLSS). It was perceived that the new government had seen NHIS as a social protection intervention better aligned with MoLSS than MoH. Most participants viewed the move to MoLSS as a disruptive and misguided action that destabilized administrative arrangements and operations of NHIMA. It disconnected the MoH from NHIMA activities, resulting in a change in the focus of the scheme and a lost opportunity to comprehensively reflect NHIMA in MoH policies developed at the time.

MoH had a dedicated directorate that was linked to NHIMA. NHIMA also with its subsections and so forth. But the problem came is, the implementation of NHIS was compromised in my view, when NHIMA was transferred to the MoLSS.—K014

Although MoH was seen as the appropriate host for NHIMA, some participants raised concerns regarding conflict of interest and achieving a separation between the purchaser of health services (NHIMA) and the main service provider (MoH). They argued that MoH’s dual role as both service provider and NHIMA overseer could compromise accountability, impartiality and the provider-purchaser split.

But if you have NHIMA, which is not independent from MoH, which is the main provider of health services. Then how can you expect NHIMA to hold MoH to account, it can’t work.—K002

##### Desiring more autonomy in decision-making by the National Health Insurance Management Authority

Participants raised a concern about technical and professional undertakings by NHIMA being overpowered by political interests. There was consensus among participants for NHIMA to operate with greater independence in implementing the NHIS. Political interference in decisions is seen as undesirable due to conflicting interests with NHIS goals. Examples cited include decisions around the benefits package.

There’s political interference in what the benefit package should look like, on who gets enrolled into NHIMA …, but it is also important to acknowledge that it has its detrimental effects.—K006

The presence of political interference at NHIMA was discerned by the participants to be partly explained by the absence of a policy that operationalizes the National Health Insurance Act to guide the future for the scheme. It was perceived that having such a policy in place would leave less room for political interference and give NHIMA clear policy direction. The NHIMA board, which was identified as the linkage between NHIMA as an institution and the central government desired to be more inclusive, with broad stakeholder representation (including local governments, researchers, public providers, and civil society) to reflect the needs and realities of the stakeholders, make more informed decisions, and increase public trust.

So, the board should be inclusive enough to bring in all the key players, those that come from the federation of employers, the labour movement among others. If they bring in a board like that it will be a bit more independent, more inclusive—K003

##### Indicating a concern about the government’s long-term commitment to the National Health Insurance Scheme

Participants emphasized the need for strong political will and sustained government commitment as critical enablers for the successful implementation of the NHIS. This included visible support for the scheme’s continuity and proactive efforts to mobilize public participation. Many perceived that government silence regarding the scheme, noting a lack of visible commitment and reluctance to recruit the NHIMA board and staff, as concerning. It was stated that this led to slow staff transitions within the new government introducing delays in full operationalization of the scheme’s governance structures. The participants urged the government to clearly articulate its vision and objectives for NHIMA. Some participants questioned the government’s commitment to sustaining the scheme, expressing concern that its future may be under reconsideration. However, they noted that potential political costs and the existence of an Act of Parliament make a policy reversal unlikely. Some participants perceived that the scheme may be viewed as a legacy of the previous administration, potentially influencing the level of political ownership and support it receives under the current government.

I have never heard the top man [the President of Zambia] talk about it [NHIS] on any platform. ….. He talks about anything, but he has never mentioned the scheme. I tell you, in four years, he has never, ever mentioned it. That to me, is a red flag. So, you begin to wonder.—K008

….. there hasn't been as much support to NHIMA by the new government as there used to be in the previous government.—K019

#### Subtheme 2: enhancing evidence-informed and transparent governance in the National Health Insurance Management Authority

This subtheme reveals a recurring concern of the perceived lack of integration of empirical evidence, transparency, and stakeholder engagement in the formulation and implementation of NHIS policies.

##### Needing improved engagement and transparency in the National Health Insurance Management Authority’s decision-making processes

Participants noted that decisions made by NHIMA were often made in isolation of key stakeholders and with limited transparency. Participants outside NHIMA voiced that they were rarely consulted in decision-making and felt they had limited influence over the implementation of the NHIS despite their willingness to be involved. The absence of timely and inclusive communication was seen as limiting stakeholder influence on critical NHIS implementation issues. Concerns were also raised about the lack of a systematic approach to consultation and revision of the health benefit package and premiums, which was viewed as contributing to delays and reduced responsiveness in policy adjustments.

NHIMA recently revised a benefit package without any involvement from the ministry [MoH], yes, you see. So, the ministry must have a say on what is in the benefit package, yes.—K012

Further, participants noted weak communication and coordination between NHIMA and MoH. Many expressed concern over NHIMA’s absence from MoH-led meetings. MoH representatives confirmed that, until recently, there was no dedicated unit to manage NHIMA-related matters.

…., we [MoH] should be aware, we should have preview information of exactly what, how they are running, how much they've raised this month, yes, you know, how much they've dispersed, gaps and everything, we don't get that.—K012

##### Highlighting gaps in evidence use within the scheme and perceptions of weak evidence integration

Participants noted that available evidence has played a limited role in NHIS implementation and is often overlooked in decision-making processes. For example, actuarial assessments’ recommendations on the contributory rate (premium) have not been acted upon.

The initial design [based on actuarial assessments] was that an employee was supposed to contribute 2.5% and the employer 2.5% towards the scheme. Altogether 5%. …. With politics involved, you know, it was reduced to 2%. [1% contribution for employer and employee]—K014

Participants mentioned that NHIMA routinely collects observational data through its core operational activities, including enrolment, provider accreditation, claims processing, and reimbursements. However, participants shared that this data remained largely unprocessed for strategic insights and underutilized for decision-making, especially during the early stages of NHIS implementation. They emphasized the need for NHIMA and government to invest more in evidence generation to inform future decisions, e.g. evidence on the impact of NHIS in relation to its objectives.

So, we have not done an assessment. Under the current Zambia Household health utilization and Expenditure survey, there was a component on the impact of NHIMA, but that study has not been done. So, I wouldn't say it has been affected because there is no evidence.—K001

### Theme 2: equity in question: winners and losers

This theme highlights the perceived differential impact of NHIS implementation across population and health provider subgroups.

#### Subtheme 1: witnessing the creation of a fractured system with well served and poorly served areas and people

The subtheme describes participants’ fear of NHIS widening health inequities, especially with access to quality health services.

Raising concerns about the emergence of a two-tier system, one for the rich and insured people and another for the poor and vulnerable.

Participants see NHIS as creating and reinforcing a stratified two-tier system disaggregated by one’s socio-economic status. Participants noted that NHIS grants members access to high-quality care in private facilities and NHIS-dedicated wards in public facilities. As a result, NHIS members receive better healthcare across both health provider types compared with non-members. Participants observed that NHIMA members are generally more well-off, while non-members tend to be less affluent. They expressed concern that NHIS may be deepening existing disparities in access to quality healthcare, widening health inequities rather than reducing them.

There is a two-tire system where there is a good health care for those who are members of NHIMA and then a very horrible one for those who are not members of NHIMA.—K011

Those kinds of inequalities were supposed to be done away with. But at the inception, we saw the creation of those [NHIMA] wards [providing care to only NHIMA members], however, the explanation given was that this was an approach meant to steer interest among people to register with the scheme.—K016

The majority of participants expressed concern that despite constituting a significant proportion of the population, individuals in the informal sector, along with the poor and vulnerable, remain under-represented within the scheme, with limited visible commitment from either NHIMA or the government to actively enrol them. NHIMA-affiliated participants noted the existence of some targeted efforts, including a pilot project enrolling a few poor and vulnerable individuals with donor support and mass media campaigns aimed at increasing enrolment among informal sector workers. However, participants noted that these measures have yielded minimal impact and emphasized the need for NHIMA to adopt more innovative and context-specific strategies to improve coverage.

But in terms of coverage, a lot of people are not covered, those that are under the social cash transfer program, these are the poorest of our society and those in the informal sector.—K004

##### Ensuring an equitable distribution of accredited facilities across urban and rural areas remains a challenge

NHIMA has accredited health facilities (both public and private facilities) across both rural and urban areas to provide services to its members. Participants expressed concern about accredited facilities being concentrated in urban areas. Given the perception that private facilities offer higher quality care, the participants expressed that rural residents face limited access to such services unless they travel to urban centres, an option that increases the cost and burden of seeking care.

Accreditation was largely focused along the line of rail. For communities that are in far-flanked areas [rural areas], accreditation was rather slow. So, you find that much of the accredited institutions still remained within Lusaka, Copperbelt. For Southern province, these are within the line of rail—K016

Participants raised questions regarding the existence, clarity and transparency of NHIMA’s accreditation strategy and process, particularly considering its recent inclusion of lower-level health facilities. This approach appeared to diverge from the original intent of accrediting only facilities offering secondary and tertiary care.

##### Setting fair premiums remains difficult, especially for individuals with little or no income

Participants desire that NHIMA set fair premiums for all people, with everyone contributing something to the scheme. However, they acknowledged the difficulty in determining what is fair in terms of premiums, especially for people in the informal sector who have large variations in their incomes that are difficult to trace or confirm.

But there's a large section of people that are outside any payroll system. Their income is varied, others have a lot of money, others very little, very poor. Now the question is, how do you really determine how much they pay?—K005

The participants indicate that the affordability of the premiums is a challenge for a considerable proportion of the population, with some people being unable to pay the set premiums, especially for the low- to no-income earners. Some participants noted that the intermittent payment of premiums, especially when patients seek care, may be reflective of the seasonal incomes for the households and not adverse selection. It was also mentioned that for the poor and vulnerable that receive a social cash transfer, a small amount of that money should be remitted to the scheme as a premium.

When you look at the population of Zambia, the economic status welfare, this [NHIS] is a difficult thing to implement. Look, majority of the people genuinely struggle with premiums. So even when you say you are paying twenty kwacha [$1]. There will be a lot of people that will struggle to pay that amount.—K007

#### Subtheme 2: needing to improve distribution of the National Health Insurance Scheme benefits among the public and private providers

This subtheme concerns the perception that the allocation of NHIS resources is skewed towards private providers, prompting concerns about underfunding in the public healthcare sector.

##### Seeing disproportionate and inefficient benefit from the National Health Insurance Scheme resources by type of provider

Participants highlighted a structural imbalance in the allocation of NHIS resources, with private healthcare providers receiving a disproportionately larger share compared with public providers, despite their limited presence and coverage across the country. This was attributed, in part, to the differential tariff structure applied to providers where private providers charge higher fees for equivalent health services.

But the difference is in the pricing that is there. I will give you an example, on the capitation for OPD, in the public hospital, you probably get 200 kwacha on the first visit by the patient, on the second visit the hospital will claim 150 kwacha. The 150 can be claimed for two visits. Then from there if the patient has a chronic condition, and they come in every now and then, then the fee goes down to 35 kwacha per visit. But when you go to our colleagues in the private sector, for each OPD visit, they get about 700 Kwacha.—K021

Participants noted that NHIMA members’ freedom to choose providers has shifted patients towards private facilities, driven by perceptions of higher quality care. Public facilities often refer patients to private providers for diagnostics or medications when services are unavailable or equipment fails. These referrals, combined with provider choice, increase claims and resource use by private providers under NHIS. Participants suggested prioritizing NHIS resources for public facilities, as they serve a larger share of the population.

##### Managing provider dissatisfaction and mitigating risks to scheme performance

Some participants, particularly from NHIMA, noted recent tariff revisions that bundled health services and specified refill and follow-up intervals for chronic conditions. Concerns were raised regarding the perceived downward adjustment of tariffs that did not adequately reflect the prevailing economic conditions and were below the actual cost of care. As a result, some providers were said to be adjusting service inputs to remain within reimbursement limits or, in some cases, requesting additional payments from patients to maintain service quality.

Everything else has gone up, but the NHIMA tariffs have not, in fact, the NHIMA tariffs have been curtailed from January this year [2025], … Now, when you go to a pharmacy asking them to supply you medication on NHIMA, most pharmacies are now refusing. So, it's double tragedy for the patient. ….. So, what is happening, you have to pay out of pocket, or you don't get treated.—K015

### Theme 3: pointing at the need for financial sustainability of the National Health Insurance Scheme

This theme reflects perceptions towards the implementation of the NHIS focusing on the critical importance of ensuring financial sustainability of the scheme.

#### Subtheme 1: Continuing to implement strategies to tackle health insurance inefficiencies

This subtheme reflects participants’ emphasis on the need for NHIMA to become financially self-reliant, expressing concern over a growing financial deficit driven by rising healthcare demand, limited revenue, and reliance on unstable donor funding.

##### Desiring that the National Health Insurance Scheme becomes self-reliant and operates as a financially independent health insurance scheme

Participants were convinced that NHIMA is experiencing financial hardship, where the total amount collected in premiums is lower than the amount of money paid out in reimbursement of providers. It was mentioned that NHIMA has tried to lobby for additional funding from different sources but realized funds were deemed insufficient including those from the Global Fund. Participants expressed concern that rising demand from noncommunicable diseases (NCDs) and population growth could worsen NHIMA’s financial deficit if unaddressed. They agreed on the need for innovative, sustainable revenue strategies, emphasizing financial self-sufficiency over reliance on external funding viewing donor support as unreliable.

NHIMA is struggling financially. It is currently collapsing because it's taken into its mouth more than what it can manage to chew. Instead of focusing on particular areas to serve and reduce the gap on the National resource envelope, it undertook to do everything, which was not necessary. Now its expenses have superseded the collections in premiums. At the time when it was being conceived, it was feasible but not anymore.—K010

##### Addressing performance gaps in the National Health Insurance Management Authority to drive system improvements

Participants expressed concerns about inefficiencies in NHIMA’s implementation of NHIS. Key issues included prioritizing nonessential services like vision aids and dental care offered in commercial malls, where aggressive marketing was believed to drive unnecessary demand and increase costs. They also noted duplication of services, such as reimbursing primary care already funded by the MoH and overlapping roles in the accreditation of providers (typically done by the Health Professions Council of Zambia) and benefit package development (executed by the MoH). Outsourcing to underperforming contractors with conflicting interests was another concern. Participants urged NHIMA to adopt priority setting and strategic purchasing, return outsourced functions in-house, and address inefficiencies urgently to reduce financial deficits and improve sustainability.

But from the first day when the door opened, you know, there were optical services, quite a number of items. So, it didn't take long before they were overwhelmed by the claims because some providers actually took advantage of that.—K019

Participants stated that the scheme lacks a functioning gatekeeping mechanism; hence, NHIMA members can access services in any NHIMA-accredited health facility, whether private or public. Participants pointed out that this had consequently collapsed the referral system. They indicated the need for NHIMA to develop a functional gatekeeping system to tame unnecessary access to health services, thus reducing NHIMA expenditure.

In our system, somebody from home can go directly to UTH [National referral hospital]. So, the lack of gatekeeping mechanisms is having an impact on the amount spent by NHIS.—K008

##### Tackling moral hazard and adverse selection among private providers, people in the informal sector and rural areas

Participants identified certain NHIMA policies as inadvertently fostering adverse selection and moral hazard. While measures such as abolishing the buyback clause and deregistering non-compliant providers have been introduced, these challenges persist, particularly due to weak post-accreditation monitoring. Moral hazard (supplier-induced demand) was perceived as prevalent mostly among private providers. To mitigate this, participants recommended enhancing transparency in billing processes, including encouraging patients to verify claims before submission for reimbursement.

But what took a turn is when the private sector started, in my opinion, started to game the system. The scheme didn't react fast enough to put safety measures against over utilization, provider induced demand, fraud.—K008

#### Subtheme 2: managing expectations: rising scepticism over the National Health Insurance Scheme’s sustainability

This subtheme describes participant’s growing scepticism about the scheme’s viability, citing a disconnect between its intended design and practical implementation.

##### Acknowledging a shared vision amidst growing scepticism about the National Health Insurance Management Authority’s goal attainment

Participants agreed that the NHIS was established to advance UHC, with key goals including financial protection, improved access, and better quality of care. However, many emphasized that the scheme’s primary driver was to mobilize domestic resources to support public health service delivery amid fiscal constraints. Enhancing health system efficiency was seen as a secondary aim. These views reflect a perceived hierarchy of objectives, with resource mobilization viewed as the most immediate and pragmatic priority underpinning broader UHC aspirations.

I think is that NHIMA was put in place to raise revenue for the government, you know, to improve financing for coverage of health care services, as well as, as one of the measures also to just promote universal health coverage.—K017

Participants confessed that they were enthusiastic about the scheme’s aims at the start, but over time, growing scepticism has emerged regarding their feasibility. This shift in perception was attributed, in part, to disappointment that key elements envisioned during the design phase and the intended operational model had not been fully realized in practice.

##### Challenging start of the National Health Insurance Scheme with limited resources, support and trust from stakeholders

Participants acknowledged that the scheme encountered significant challenges during its early implementation stages. It was stated that NHIMA was established without enough resources available to fully operate, as no seed money or start-up funding was made available by the government.

First of all, the implementation was very difficult. Extremely difficult in the sense that there were no adequate resources available to start NHIMA from the resource envelope to human resources, offices, equipment… You can imagine that you are starting, and you don't even have an office. You don't have a vehicle. You are using your own vehicle, your own stationery, your own printing.—K013

It was perceived that the country’s economic status at the start of the scheme was equally challenging, grappling with debt without money to invest in the scheme.

##### Highlighting the gaps in the National Health Insurance Scheme sensitization

Participants acknowledged NHIMA’s efforts to raise awareness about NHIS through media and hospital-based registration points. However, they noted that many people and providers still lack understanding of health insurance and how it functions. Current outreach was seen as limited, leaving room for misinformation. They also observed that NHIS communication focuses mainly on benefits, with little mention of member responsibilities. Participants noted that NHIMA members often expect preferential treatment and visible recognition in hospitals, leading to unrealistic expectations compared with non-members.

NHIMA created lots of expectations among the general public when it opened doors.—K019

##### Balancing comprehensiveness and costs in the National Health Insurance Scheme benefits

The participants perceived the scheme as being generous due to its extensive health benefit package, covering a long list of services, allowing contributing NHIS members to register up to six beneficiaries, having exemptions for some population subgroups (like pensioners) and a low premium (2% of one’s income). The health benefit package was said to be broad covering primary, secondary and some of the tertiary level healthcare services at any NHIMA-accredited health facility.

They contribute 50 kwacha [$2] for a family of six. So already the collection for much less than the potential demand that you have. Which makes you already vulnerable to bankruptcy because your risk is not minimized by the number of people you are covering.—K003

##### Observing improved provider revenues and quality of care

Participants acknowledged NHIS’s positive impact on resource availability and care quality, despite remaining challenges. It was mentioned that increased resources came through NHIMA reimbursements and a seed fund for quality improvements. Participants acknowledged that public facilities showed notable progress, with NHIMA-enrolled patients experiencing shorter wait times, better access to diagnostics, and cleaner environments. These changes reflect enhanced service delivery and improved patient experience under NHIS.

When NHIMA started, they provided a seed fund called CAP [Claims Advance Payment]. …So, from CAP, hospitals innovated, they hired additional number staff, procured drugs. From the assessment conducted in 2022, there was 80% level of satisfaction.—K001

## Discussion

We investigated the perceptions of stakeholders towards the implementation of the NHIS describing the enablers and challenges faced by the scheme. The study reveals a complex and evolving policy landscape shaped by both structural and contextual factors. The findings of this study suggest a substantial divergence between the scheme’s original design and its execution, with political dynamics playing a central role in shaping governance and decision-making processes. Participants highlighted perceived inequities in benefits distribution across population subgroups and health providers, alongside concerns about the scheme’s financial sustainability. Limited political commitment, constrained use of evidence, and on-going political interference were seen by stakeholders as key barriers to effective implementation. Despite these challenges, the stakeholders widely acknowledged the scheme for its generous benefits and strong public support, highlighting its potential to advance health system goals.

### Politics can make or break the scheme

The implementation of Zambia’s NHIS is perceived to be widely influenced by politics, mirroring trends observed in health policy processes across diverse country contexts (Hanson et al. 2022, Buse et al. 2024). Participants described how political interference in the governance and decision-making structures of NHIMA has compromised the scheme’s autonomy and operational effectiveness. These findings align with existing literature suggesting that health policy implementation is often driven more by political expediency than by empirical evidence. For instance, Ansah et al. (2024) highlight how short-term political interests frequently override long-term health objectives, leading to fragmented policy development and implementation gaps. Similar dynamics have been documented in Ghana, Nigeria, and Kenya, where health insurance reforms have been subject to political bargaining, with limited protection of technical agencies from political pressure (Alhassan et al. 2016, Oraro-Lawrence and Wyss 2020, Ansah et al. 2024, Chukwuma and Obi 2025). The reported political interference in Zambia’s NHIMA may explain the gap between what the NHIS was originally intended to implement and what has been implemented, such as the difference between the planned universality of the scheme and its current level of coverage.

The perceived limited integration of empirical evidence into NHIS decision-making in Zambia reflects broader challenges observed in similar settings. As highlighted by Liverani et al. (2013), institutional weaknesses, donor influence, and the politicization of health policy often constrain the use of evidence in LMICs. In the Zambian context, participants perceived that technical evidence, such as actuarial assessments and routine data, was frequently sidelined due to competing political agendas. This marginalization of evidence was seen as both a symptom and a consequence of political interference, contributing to growing scepticism about the scheme’s ability to achieve its intended goals. It also helps explain participants’ calls for greater autonomy in NHIMA’s decision-making processes.

Our findings underscore the need for stronger institutional frameworks that foster transparency, stakeholder engagement, and systematic evidence use. The literature suggests that insulating health policy from undue political influence requires not only political commitment but also enhanced capacity for evidence-informed policymaking and mechanisms for accountability (Liverani et al. 2013, Ansah et al. 2024). Although the participants decried political interference at NHIMA, they emphasized that a robust political commitment is essential for the scheme’s success as also reported by Cashin and Dossou (2021). Public endorsement of the scheme by political leaders could be a key strategy to build public support and encourage new members to enrol. In this regard, Witter et al. (2025) suggest that insurance agencies can better navigate political dynamics by conducting political economy analyses to anticipate and strategically manage contextual challenges and opportunities during implementation.

Despite the above constraints, the study also identified important perceived enablers of NHIS implementation. These include the scheme’s legal foundation, growing analytical capacity within NHIMA, and strong public support. The statutory basis of the NHIS was viewed as a safeguard against abrupt policy reversals that usually come with change in governments, while the recruitment of data analysts signalled an emerging institutional capacity to leverage evidence for decision-making. Moreover, the scheme’s popularity among beneficiaries (NHIS members), who valued their experience of financial protection and improved access to care, was seen as a source of political legitimacy and resilience as reported by Osei Afriyie et al. (2025). Similar enabling factors have been documented in Rwanda’s community-based health insurance and Ethiopia’s insurance reforms, where legal backing, data use, and public trust have contributed to implementation success (Cashin and Dossou 2021, Habte et al. 2022).

### Equity in question: winners and losers

The NHIS was launched as way for Zambia to move towards UHC by expanding access to hospital-level care while offering financial protection (Republic of Zambia 2018). This understanding was well reflected among the participants’ views. However, participants in this study perceived that the scheme has inadvertently exacerbated inequities in access to quality care. Those who are wealthier and reside in urban areas were seen as benefiting disproportionately, while poorer and rural populations continue to face barriers to accessing high-quality services. This perception aligns with findings from Barasa et al. (2021), who observed that health insurance schemes in sub-Saharan Africa tend to be pro-rich where the majority of the health insurance members are well-off and the majority of the non-members are the less well-off. Such inequities risk undermining the legitimacy of the NHIS and eroding public trust, both of which are critical to the long-term success of any health insurance model (Kutzin 2001). Participants also described the emergence of a two-tiered health system, where NHIS members, primarily formal sector workers, access better quality services, particularly in private facilities, while non-members rely on under-resourced public providers. Similar patterns of stratified care have been documented in Ghana, Nigeria, and other sub-Saharan African countries, where insurance schemes have reinforced disparities in service quality between urban and rural areas, and between insured and uninsured populations (Alhassan et al. 2016, Christmals and Aidam 2020, Barasa et al. 2021, Watson et al. 2021).

In addition to inequities among beneficiaries, participants highlighted concerns about the distribution of NHIS resources across types of health providers. Private providers, who serve a smaller and more affluent segment of the population, were perceived to receive a disproportionate share of reimbursements compared with public providers, who cater to the majority. This imbalance was seen as potentially weakening the public health system and distorting provider incentives. Comparable concerns have been raised in Kenya, where the National Hospital Insurance Fund has been criticized for favouring private providers through higher reimbursement rates, raising questions about efficiency and fairness (Barasa et al. 2018).

Another equity concern raised by participants was the limited inclusion of the informal sector, poor, and vulnerable populations, groups that constitute the majority of Zambia’s population. Despite the scheme’s UHC aspirations, these populations remain largely excluded, echoing challenges faced by other LMICs (Barasa et al. 2021). In Ghana, for instance, informal sector enrolment has remained low due to affordability constraints and weak enforcement mechanisms (Alhassan et al. 2016). Ethiopia’s community-based health insurance has made more progress in reaching these groups, though its sustainability is challenged by heavy reliance on government and donor subsidies (Mulat et al. 2022). Evidence from multiple countries, including Kenya, Indonesia, and Burkina Faso, suggests that government subsidies for premiums can significantly improve enrolment among underserved populations, offering a potential pathway for Zambia to enhance equity within its NHIS (James and Acharya 2022).

### Pointing at the need for financial sustainability of the National Health Insurance Scheme

Despite the popularity of the scheme among enrolees, participants cautioned that the scheme’s long-term viability is undermined by low population coverage, operational inefficiencies, and a mismatch between its generous benefits and limited revenue base. The sustainability of any insurance scheme hinges on broad risk pooling, which requires high enrolment (Carrina and James 2004). However, Zambia’s NHIS covers only about a quarter of the population (National Health Insurance Management Authority 2025), raising concerns about its legitimacy and financial resilience. This challenge is not unique to Zambia; contributory schemes in many LMICs struggle to include informal sector workers due to income variability, weak administrative systems, and affordability barriers (Barasa et al. 2021, Yazbeck et al. 2023). For example, countries like Kenya (Nungo et al. 2024), Nigeria, Liberia, Mali, Senegal, Togo, Zimbabwe, and Sierra Leone have a coverage of <20% of the population (Barasa et al. 2021). Findings echo these concerns, noting that the exclusion of sections of the population undermines the scheme’s solidarity principle and sustainability.

In contrast, countries such as Rwanda (79% coverage; Barasa et al. 2021), Ghana (58% coverage; Barasa et al. 2021), Peru (99% coverage; The World Bank 2023), Colombia (99% coverage; Brun Vergara et al. 2023), and China (97% coverage; Lee et al. 2022) have achieved higher coverage through a combination of government subsidies and diversified financing strategies. Participants suggested that Zambia could follow similar pathways by increasing public financing for vulnerable groups, as evidence shows that subsidies are more effective than information campaigns in boosting enrolment (Adebayo et al. 2015, James and Acharya 2022).

This study also highlighted inefficiencies within NHIS operations, including overlapping functions, high administrative costs, and duplication of services. These issues mirror findings from other settings and point to the need for institutional reforms (Brikci et al. 2024, Nungo et al. 2024, Olyaeemanesh et al. 2024). Embracing priority-setting tools and health technology assessment was proposed as a way to enhance value for money and guide evidence-informed purchasing decisions (Olyaeemanesh et al. 2024).

The scheme was praised for improving quality of care, particularly through increased provider resources and infrastructure. This finding contradicts evidence in similar countries where health insurance was found to hardly have any impact on the quality of care (Spaan et al. 2012, Erlangga et al. 2019, Osei Afriyie et al. 2023a). The impact of health insurance on quality of care could be influenced by the reimbursement mechanisms used in the scheme where the use of capitation is associated with declining quality of care while fee for service is associated with an increase in quality of care (Erlangga et al. 2019). Despite the compliments of the scheme, its generosity was also seen as a liability. The broad benefit package, inclusion of multiple dependents, and low premiums were viewed as financially unsustainable. NHIMA’s recent cost-containment measures, such as tariff reductions, have triggered dissatisfaction among providers and unintended consequences, including informal co-payments and supplier-induced demand. These dynamics risk eroding financial protection and quality of care, ultimately threatening the scheme’s core objectives.

Social health insurance, which Zambia’s NHIS was intended to be an example, has long been promoted as a reliable pathway for countries to advance towards UHC (Carrina and James 2004). A core principle of social health insurance is broad population coverage, which is why having a substantial proportion of the population in the formal sector is often considered a prerequisite for establishing such schemes. The experience in Zambia, as reflected in stakeholders’ perceptions, highlights several challenges which could be partly arising from the country’s large informal sector employment relative to its formal sector. This raises important questions about whether similar LMICs with predominantly informal sector employment should rely on social health insurance as a strategy for progressing towards UHC, particularly when they struggle to include individuals who are unable to pay premiums. Click or tap here to enter text.

Overall, the results of this study point to the challenges experienced by NHIS in working towards UHC. Contributory health insurance schemes in LMICs have faced criticism for excluding vulnerable populations, failing to mobilize additional resources, and undermining health system efficiency (Yazbeck et al. 2023). Zambia’s NHIS reflects many of these concerns, with participants highlighting the exclusion of informal sector workers and the poor, persistent inefficiencies, and questions around financial sustainability. However, exceptions such as Rwanda, Ghana, Gabon and Peru demonstrate that progress is possible when schemes are supported by strong political will, diversified financing, and inclusive design (Yazbeck et al. 2023). This study contributes to the ongoing discourse on what works in LMIC contexts. Participants emphasized the need to expand coverage by enrolling left-out groups, which will require subsidized premiums and targeted government support. Such measures are essential not only for equity but also for the financial viability of the scheme and its contribution to UHC.

To enhance the trustworthiness of the findings, we applied Lincoln and Guba criteria for assessing qualitative rigour (Amankwaa 2016). The authors, particularly J.K. and F.M., spent considerable time in the field to familiarize themselves with the study context and understand the realities of the NHIS. Peer debriefings were conducted after each interview, allowing the interviewer to receive feedback from co-authors and reflect on their role in the data collection process. In addition, personal reflective notes were kept throughout the research process to support neutrality. The findings of this study, therefore, may be useful and applicable to other settings with a similar context to Zambia.

### Limitations

First, the data collection coincided with media coverage of NHIMA’s financial challenges, which may have influenced participants to emphasize implementation difficulties. This was countered by the existence and use of the interview guide enabled that all relevant aspects of the interview to be covered. Second, some informants were not involved in the early stages of NHIS, potentially limiting insights into its initial rollout. To mitigate this, we included individuals previously engaged in the scheme’s inception. Third, while key policy stakeholders were well represented, certain actors, such as representatives from MoLSS and parliament, were not interviewed which may have led to missed perspectives. The sampling frame used for this study, aimed for variation among the stakeholder however some the targeted participants were unavailable during the interview period and thus less represented in the final sample. Therefore, the distribution of participants across stakeholder categories was uneven, with some groups, such as the MoH and academicians, being more represented than others, like civil society and funders which may affect the breath of the participant experiences captured by the study. Nevertheless, the diversity of participants included in the study likely captured the core themes relevant to NHIS implementation as found through member checking where participants reviewed and affirmed that the interpretations accurately reflected their experiences and perspectives, thereby validating the authenticity of the study results. Lastly, most participants were based in urban areas, which may have limited the inclusion of perspectives from those living in more remote regions. While this geographic imbalance could have constrained the diversity of viewpoints, it is unlikely to have substantially influenced the overall findings given the study’s focus on national-level stakeholder perceptions.

## Conclusion

The implementation of Zambia’s NHIS is complex and faces substantial challenges. While the NHIS is perceived as a promising initiative for expanding access to health and enhancing financial protection, stakeholders highlighted persistent challenges ranging from limited enrolment among the poor, vulnerable and informal sector populations as well as concerns about long-term financial sustainability. Addressing these issues will require targeted structural and operational reforms, including expanding the contributor base, aligning benefits with revenue, and improving efficiency through strategic purchasing and institutional accountability. Despite the constraints, the stakeholders acknowledged that scheme enjoys public support, offering a foundation for increased enrolment and advancing reform efforts. Although this study represents diverse categories of stakeholders, expanding participation among under-represented groups would further enrich understanding of the issues identified.

## Supplementary Material

czag074_Supplementary_Data

## Data Availability

The data underlying this article are available in the article and in its online supplementary material.

## Reflexivity statement

The study was conducted by a team of eight researchers of which half are female. The team is comprised of two early career researchers in health economics and financing, five senior researchers in health economics and health systems, and one senior researcher in social medicine and qualitative methodology. The team has long research experiences within the Zambian context in health policy analysis and impact evaluation.
