# Supplementary material for: A qualitative study of stakeholders’ perceptions towards the implementation of the National Health Insurance Scheme in Zambia: equity and financial sustainability in question
Source: Health Policy Plan. 2026 May 25;41(7):1088–101. doi: 10.1093/heapol/czag074 (PMC13481678; doi:10.1093/heapol/czag074)
Supplement: Supplementary file 1 [file czag074_supplementary_data.docx]

## Supplementary material

**Supplementary material 1: Details about study participants**

The central government participants were included because they oversee the implementation of the regulation. Representatives from the Ministry of Health (7 participants) were included because the ministry regulates the health sector and is the current host of NHIMA. Local government representative (1 participant) was included to provide experiences of lower-level governments. The scheme managers, NHIMA (4 participants) were part of the respondents because they oversee the scheme. Health practitioners’ representatives through their associations and councils were included (2 participants). Academicians who provide the research evidence on health financing in the country were also included (5 participants). Funders were included to provide perspectives of funders that support NHIMA or government in line with the topic (1 participant) and civil society (1 participant). A total of 21 participants were included in the study.

**Supplementary material 2: Interview guide**

1. Can you please describe your professional role and how that role relates to NHIS/NHIMA?
2. Can you please describe your perception of the motives behind introducing NHIS/NHIMA?
3. Can you please, in your own words, describe your perception of the implementation of NHIS so far?
4. Can you please share your understanding of the organizational structure of the scheme?
5. Let's talk about enrollment with NHIMA. What is your perception of enrollment with NHIMA?
6. Now let's move over to funding. What is your perception of how NHIMA is funded?
7. Let’s then discuss the benefit of NHIMA membership. Can you please describe your perception of the current NHIMA health benefit package?
8. Can you please share your understanding of how NHIMA is spending the money it collects?
9. On accreditation of health providers, what is your perception about the accreditation process of the providers by NHIMA?
10. Can you please describe your perception about the amount of money paid to the providers for the services offered?
11. Can you please share your understanding on how the NHIS implementation relates to UHC?
12. Is there anything more that you would like to add that we have not talked about?
